# Multivariate analysis of factors associated with spinal cord area in single-door cervical laminoplasty with miniplate fixation

**DOI:** 10.1186/s12891-021-04773-w

**Published:** 2021-10-15

**Authors:** Ke-rui Zhang, Yi Yang, Hao Liu, Bei-yu Wang, Chen Ding, Yang Meng, Xin Rong, Ying Hong

**Affiliations:** 1 Department of Orthopedic West China Hospital, Sichuan University, No. 37 Guo Xue Xiang, Chengdu, 610041 Sichuan China; 2 Department of Operation Room, West China Hospital, Sichuan University, No. 37 Guo Xue Xiang, Chengdu, 610041 Sichuan China

**Keywords:** Spinal cord area, Spinal canal area, Single-door cervical laminoplasty, Door shaft position

## Abstract

**Objectives:**

To explore the factors associated with the increased spinal cord area in single-door cervical laminoplasty (SDCL) with miniplate fixation.

**Methods:**

A retrospective study enrolled 83 patients underwent SDCL with miniplate fixation and the patient characteristics such as age, gender, tobacco use, alcohol use, diabetes mellitus, hypertension, diagnosis, operative level, etc., were obtained. The opening angle, door shaft position and spinal canal area of the patients were measured after surgery. The sagittal canal diameter (SCD), the C2–7 Cobb angle, the cervical curvature index (CCI), the range of motion (ROM) and the spinal canal area were measured before and after operation. The increased cervical spinal cord area was also measured before and after surgery, and the correlation between the above indicators and the increased cervical spinal cord area was studied through Pearson’s correlation analysis and multivariate logistic regression analysis.

**Results:**

There were 34 patients in small spinal cord area increment group (SAI group), 29 patients in middle spinal cord area increment group (MAI group) and 20 patients in large spinal cord area increment group (LAI group). The preoperative diagnosis(*P* = 0.001), door shaft position (*P* = 0.008), preoperative spinal canal area (*P* = 0.004) and postoperative spinal canal area (*P* = 0.015) were significant different among the 3 groups. The multivariate analysis showed that the preoperative diagnosis (OR = 2.076, *P* = 0.035), door shaft position (OR = 3.425, *P* = 0.020) and preoperative spinal canal area (OR = 10.217, *P* = 0.009) were related to increased spinal cord area.

**Conclusions:**

The preoperative diagnosis, door shaft position and preoperative spinal canal area might be associated with increased spinal cord area after cervical laminoplasty with miniplate fixation. Preoperative symptoms are mostly caused by compression of the spinal cord, so spinal cord area enlargement can bring a better recovery in patients alongside long-term. Spine surgeons should pay more attention to the accuracy of the preoperative diagnosis, the preoperative measurement of spinal canal area and the door shaft position during the operation.

## Introduction

Single-door cervical laminoplasty (SDCL) has been widely used for patients with ossification of the posterior longitudinal ligament (OPLL), multiple cervical discs herniation (MCDH) and degenerative cervical spinal canal stenosis (DCSS) [1]. The laminoplasty is to incise completely through one lamina of a vertebral arch with creation of a trough in the contralateral lamina; the vertebral arch is then opened like a door, with the trough acting as a hinge; performed to relieve compression of the spinal cord or nerve roots, which fixed by suture suspensory or miniplate, etc. Some studies found that mini-plate fixation was superior in reducing the incidence of surgical complications, which is an increasing popular surgery and a good choice for patients [2]. The purpose of cervical laminoplasty is to indirectly relieve the compression of the spinal cord by posterior enlargement of the spinal canal, which enables a better recovery for patients, as well as a long-term effectiveness of the surgery [3, 4].

In recent years, there are extensive studies to explore the relationship among opening size, door shaft position, opening angle, sagittal canal diameter (SCD), spinal canal area and axial pain after SDCL [5–8]. However, most of the preoperative symptoms are caused by compression of the cervical spinal cord [9]. The increased spinal cord area after open-door surgery can indirectly reflect the effect of decompression, which is a problem worthy of our attention. But few studies focused on the increased spinal cord area after surgery, especially for the cervical laminoplasty with miniplate fixation [10]. The objective of the current study was to observe the increased spinal cord area and to elucidate the potential factors associated with it after SDCL with miniplate fixation.

## Methods

### Patient data

This was a retrospective study approved by the Ethical Committee of West China Hospital of Sichuan University. Patients were selected from November 2011 to June 2018. All patients had given the informed consent to allow their information to be used with research purposes.

In the present study, all of the patients included in the study were diagnosed with OPLL, MCDH or DCSS according to their clinical manifestations and results of x-rays and computed tomography (CT) and magnetic resonance imaging (MRI) scans before SDCL with miniplate fixation. The conservative treatment was used for at least 3 months and all cases were admitted to our hospital for surgical treatment. MRI indicated cervical disc herniation or spinal stenosis involving at least 3 intervertebral levels and spinal cord compression. Exclusion criteria was as follows: 1) trauma, tumor or infection at any cervical level, 2) deformity, 3) no available CT or MRI data, 4) insufficient CT or MRI data (not fully covering C2 to C7 vertebra), 5) slice thickness or slice increment larger than 1 mm, 6) the patients who received suture suspensory fixation or skipped miniplate fixation. All surgeries were carried out by a single surgeon and all the patients were followed for at least 6 months.

### Surgical technique

After receiving general endotracheal anesthesia, the patient is positioned prone with the head secured in a Mayfield 3-pin head-holder. The head and neck are held in slight flexion. A midline cervical incision is made to expose the laminae, spinous processes, and medial facet joints from C2 to C7. The ligaments are cut between C2 and C3 and between C7 and T1. The spinous processes are then amputated at their bases from C3 to C7. The opening side is made on the side with more symptoms. On the open side, a trough by completely removing the dorsal cortex, the cancellous layer, and the ventral cortex with a burr along the medial margin of the facet joints is created. Once the open side is completed, a hinge is created by making another trough on the other side. The dorsalcortex and the cancellous bone are removed, but the ventral cortex is attempted to preserve to maintain an incomplete fracture. The lamina is opened carefully with a Penfield dissector or similar instrument, and the adhesions of dural mater, ligamentum flavum, and laminae are divided. A gelatin sponge and epinephrine-containing cotton pieces can be used to decrease hemorrhaging of epidural veins. For the mini-plate fixed laminae, the appropriately sized laminoplasty plate for each level is selected using the bone trials. The Centerpiece mini-plate (Inc., USA) is inserted by fitting the cut edge of the lamina into the laminar shelf of the plate, then seating the lateral portion of the plate down onto the edge of the lateral mass. The drill bit is used to make 2 screw holes on the lateral mass and then insert two 7-mm screws to anchor the plate to the lateral mass. The 1-mm drill bit is used to drill 2 laminar holes with 2–3 mm depth, into which 5-mm screws are inserted. The other laminae are fixed using the Hirabayashi classic open-door laminoplasty techniques, where sutures are placed through the facet capsule on the hinge side and through the spinous processes to help maintain cervical canal expansion [11]. Antibiotics are administered 24–48 h after the surgery and all the patients are encouraged to perform neck exercises with a soft collar, within their pain tolerance, beginning 1 week after surgery.

### Definition of variables

Potential variables including age, gender, tobacco use, alcohol use, diabetes mellitus, hypertension, diagnosis, operative level, intraoperative blood loss, operative time, duration of follow-up, duration of postoperative hospital stay, preoperative duration of symptoms and postoperative drainage volume were obtained by retrospective reviews of medical records in our hospital.

### Radiography evaluation

**Fig. 1:**
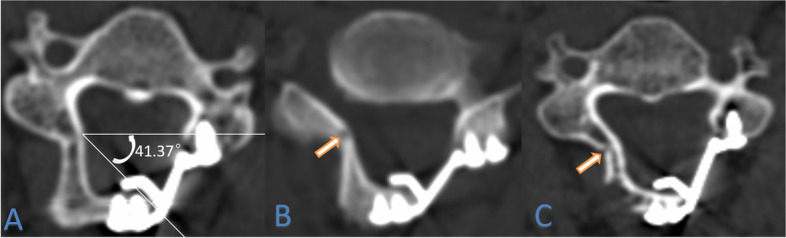
Measurement of opening angle (**A**), and door shaft position (**B**, **C**). The door shaft position is in the inner edge of the Lateral Mass, which is further outside than the side opening bone slot, this is defined as the wide opening group (**B**). The door shaft position is located in the lateral quarter of the lamina, which is defined as the narrow opening group (**C**)

**Fig. 2:**
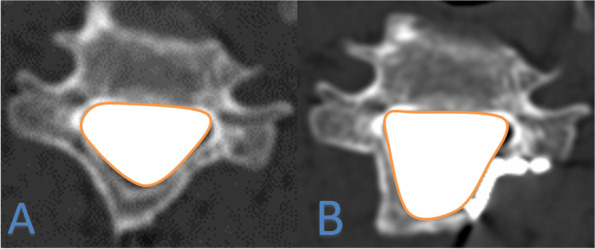
The spinal canal area before and after the operation (**A**, **B**)

**Fig. 3:**
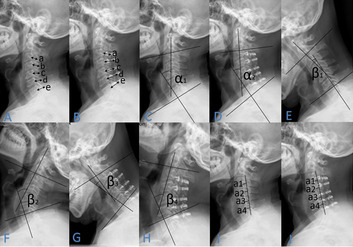
On the lateral radiograph of cervical spine, we measured the SCD of the C3-C7 segments and used the mean SCD [(a + b + c + d + e)/5] to evaluate the space of spinal canal before and after the operation (**A**, **B**), the C2–7Cobb Angle before and after the operation (**C**, **D**), the ROM before operation (β1 + β2) and the postoperative (β3 + β4) (**E**, **F**, **G**, **H**), the CCI before and after the operation, CCI = (a1 + a2 + a3 + a4)/A (**I**, **J**)

**Fig. 4:**
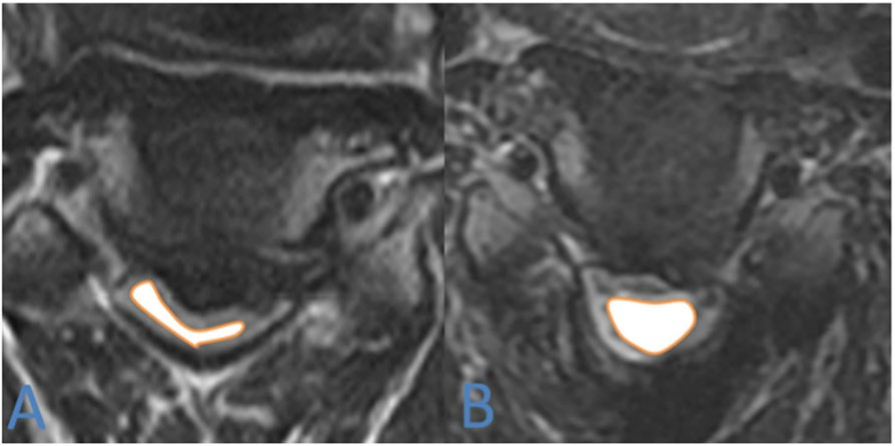
Preoperative spinal cord area at C4–5 levels(**A**), the postoperative spinal cord area at C4–5 levels(**B**)

The opening angle of C3-C7, door shaft position and spinal canal area were determined by the axial position of CT after surgery, according to the door shaft position, it is divided into wide opening group and narrow opening group (Fig. 1); the change of spinal canal area before and after operation were compare (Fig. 2); the change of SCD, C2–7 Cobb Angle, range of motion (ROM) and cervical curvature index (CCI) were measured by the lateral radiographs of cervical spine at preoperative and postoperative (Fig. 3) and the increased cervical spinal cord area were measured by MRI before and after surgery (Fig. 4). Patients with a range of 0–30% increase in spinal cord area were in the small increment group (SAI group), a 30–60% increase in spinal cord area were in the middle increment group (MAI group) and a 60 to 90% increase in spinal cord area were in the large increment group (LAI group). The spinal canal area and spinal cord area were measured using v2.1.3.250 (e2eSoft product). These data were obtained from the cross sections where the spinal cord compression was most significant.

### Statistical analysis

Statistical analysis was performed using SPSS version 19.0 software (SPSS Inc., Chicago, IL). Continuous variables with normal distribution were presented as mean ± SD. The paired t-tests were performed to detect the difference of preoperative and postoperative data. Categorical variables were compared using Pearson’s chi-square test. Continuous variables were compared using univariate analysis. According to previous studies concluded that limited decompression with an approximate 30% increase seemed to provide enough space for the spinal cord and achieve a satisfying neurological recovery [12]. In present study, MAI group (30–60% increase) and HAI group (60–90% increase) were combined into one group, the variables whose *P*-value was less than 0.05 in univariate analysis would be chosen for multivariate analysis. The multivariate analysis would be performed using binary Logistic regression analysis. The missing data would not be included in multivariate analysis. A *P*-value of less than 0.05 was considered statistically significant.

## Results

### Patient characteristics

The patient characteristics are summarized in Table 1. A total of 83 patients comprised of 65 males and 18 females with a mean age of 55.5 ± 11.8 (range from 31 to 77) years old were included. There were 34 patients including 27 males and 7 females with a mean age of 57.8 ± 10.7 (range from 43 to 76) years old in SAI group, 29 patients including 21 males and 8 females with a mean age of 53.4 ± 12.2 (range from 31 to 78) years old in MAI group and 20 patients including 17 males and 3 females with a mean age of 59.9 ± 11.5 (range from 36 to 77) years old in LAI group. The average amount of blood loss during the operation was 236 ml (range, 50–1000 ml). The mean operation time was 147 min (range, 60–240 min). The mean follow-up time was 17.2 months (range from 6 to 105). The mean length of hospital stay after surgery was 9.1 days (range, 4–21 days). Symptom duration preoperative averaged 45.8 months (range, 0.25–360 months). Postoperative drainage volume was 270 ml (range, 20–810 ml). There were no significant differences among the 3 groups in gender, age, tobacco use, alcohol use, diabetes mellitus, hypertension, operative level, blood loss, operative time, duration of follow-up, duration of postoperative hospital stay, preoperative duration of symptoms and postoperative drainage volume(*P* > 0.05). However, the difference for diagnosis was significant among the 3 groups (*P* = 0.001).

**Table 1:** Clinical Factors and Univariate Analysis of Increased Spinal Cord Area

	TOTAL	SAI	MAI	LAI	*P* Value
Gender, N (%)					0.546
Male	65	27 (41.5)	21 (32.3)	17 (26.2)	
Female	18	7 (38.9)	8 (44.4)	3 (16.7)	
Age, year	55.5 ± 11.6	57.8 ± 10.7	53.4 ± 12.2	59.9 ± 11.5	0.302
Smoking, N (%)	34	16 (47.0)	9 (26.5)	9 (26.5)	0.339
Drinking, N (%)	30	14 (46.7)	6 (20.0)	10 (33.3)	0.081
Diabetes, N (%)	21	10 (47.6)	6 (28.6)	5 (23.8)	0.729
Hypertension, N (%)	24	9 (37.5)	9 (37.5)	6 (25.0)	0.917
Diagnosis, N (%)					**0.001**
OPLL	19	6 (31.6)	8 (42.1)	5 (26.3)	
MCDH	30	13 (43.3)	11 (36.7)	6 (20.0)	
DCSS	34	15 (44.1)	10 (29.4)	9 (26.5)	
Operative level, N (%)					0.885
C2-C7	2	0 (0)	1 (50.0)	1 (50.0)	
C3-C6	8	3 (37.5)	3 (37.5)	2 (25.0)	
C3-C7	69	30 (43.5)	23 (33.3)	16 (27.5)	
C4-C7	4	1 (25.0)	2 (50.0)	1 (25.0)	
Blood loss, ml	236 ± 177	237 ± 147	190 ± 145	300 ± 244	0.098
Operative time, min	147 ± 38	140 ± 37	156 ± 44	145 ± 30	0.248
Duration of follow-up, month	17.2 ± 15.7	16.5 ± 19.4	18.1 ± 14.1	17.1 ± 11.1	0.923
Duration of postoperative hospital stay, day	9.1 ± 3.3	9.2 ± 3.7	9.1 ± 3.7	9.0 ± 2.3	0.982
Preoperative duration of symptoms, month	45.8 ± 71.6	43.0 ± 76.2	43.7 ± 56.3	53.6 ± 85.4	0.858
postoperative drainage volume, ml	270.0 ± 130.3	262.0 ± 158.1	290.0 ± 127.2	253.6 ± 93.6	0.437

*SAI* Small spinal cord area increment, *MAI* Middle spinal cord area increment, *LAI* Large spinal cord area increment, *MCDH* multilevel cervical disc herniation, *OPLL* posterior longitudinal ligament, *DCSS* degenerative cervical spinal canal stenosis

*P* < 0.05 compared to the preoperative data

### Radiography results

**Fig. 5:**
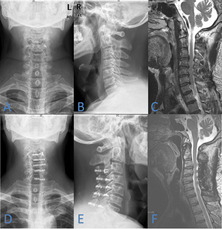
A 65 years old man patient. Preoperative X-ray plain film and middle sagittal film of magnetic resonance showed the cervical spine canal stenosis and the spinal cord compression (**A**–**C**). Preoperative X-ray plain film and middle sagittal film of magnetic resonance showed cervical canal expansive, spinal cord decompression and good miniplate fixation (**D**–**F**)

The radiography data are summarized in Table 2. The SCD, spinal canal area and cervical ROM were significantly different before and after surgery (Fig. 5). The SCD and spinal canal area increased significantly in all groups after surgery and cervical ROM decreased significantly in all groups after operation. The differences of door shaft position (*P* = 0.008), preoperative spinal canal area (*P* = 0.004) and postoperative spinal canal area (*P* = 0.015) were significant among the 3 groups. The change of the open angle, SCD, C2–7 Cobb angle, CCI, cervical ROM and other factors also did not differ significantly among the 3 groups(*P* > 0.05). And during the entire follow-up, no implant failure, mini-screw loosening, or lamina reclosure was observed in all groups.

**Table 2:** Radiography Factors and Univariate Analysis of Increased Spinal Cord Area

	Total	SAI	MAI	LAI	***P*** value
Open angle, °	39.78 ± 4.2	40.3 ± 4.5	39.6 ± 4.2	39.1 ± 3.5	0.647
Door shaft position, N (%)					**0.008**
Wide opening	38	11 (28.9)	12 (31.6)	15 (39.5)	
Narrow opening	45	23 (51.1)	17 (37.8)	5 (11.1)	
The SCD, cm					
Preop	1.64 ± 0.15	1.64 ± 0.16	1.67 ± 0.11	1.62 ± 0.16	0.497
Postop	2.51 ± 0.17	2.48 ± 0.20	2.53 ± 0.17	2.52 ± 0.12	0.528
The change	0.87 ± 0.18	0.85 ± 0.21	0.86 ± 0.13	0.90 ± 0.18	0.550
C2–7 Cobb angle, °					
Preop	12.2 ± 8.6	13.4 ± 9.2	10.6 ± 7.9	12.5 ± 8.5	0.436
Postop	11.2 ± 8.7	12.4 ± 9.4	10.4 ± 8.5	10.5 ± 8.0	0.677
The change	−1.0 ± 7.7	−1.2 ± 9.4	−0.2 ± 7.2	−1.8 ± 5.1	0.767
CCI					
Preop	0.09 ± 0.10	0.09 ± 0.11	0.08 ± 0.10	0.09 ± 0.08	0.841
Postop	0.08 ± 0.10	0.09 ± 0.09	0.07 ± 0.13	0.08 ± 0.09	0.847
The change	−0.01 ± 0.07	−0.01 ± 0.07	− 0.01 ± 0.08	− 0.01 ± 0.06	0.970
Spinal canal area, mm^2^					
Preop	191 ± 33	177 ± 30	203 ± 34	199 ± 29	**0.004**
Postop	337 ± 46	321 ± 38	353 ± 53	343 ± 37	**0.015**
The change	146 ± 29	143 ± 28	150 ± 30	145 ± 29	0.678
ROM, °					
Preop	43.7 ± 11.6	42.9 ± 11.7	45.5 ± 11.0	42.4 ± 12.6	0.907
Postop	30.8 ± 11.2	31.0 ± 10.9	33.1 ± 10.5	27.1 ± 12.3	0.176
The change	−12.9 ± 9.7	−11.9 ± 10.2	−12.3 ± 10.0	−15.3 ± 8.4	0.299

*SAI* Small spinal cord area increment, *MAI* Middle spinal cord area increment, *LAI* Large spinal cord area increment, *SCD* sagittal canal diameter, *CCI* cervical curvature index, *ROM* range of motion

*P* < 0.05 compared to the preoperative data

### Classification statistical results

Because the pathology of the OPLL is indeed different from the DCSS and MCDH, the patients who diagnosed with OPLL and non-OPLL (DCSS and MCDH) before surgery was classified into two groups and detailed statistical supplements was performed (Table 3). According to the results of statistical analysis, in addition to the preoperative CCI was statistical difference between OPLL and non-OPLL groups (*P* < 0.05), which presented the curvature of preoperative lordosis in OPLL group is larger than that of the non-OPLL group, the other preoperative anatomical parameters, the operation time, blood loss, and the postoperative drainage volume were no statistical difference between the two groups (*P* > 0.05). Due to the existence of osteophytes in OPLL, the above statistical results show that the SCD, spinal canal area and spinal cord area are smaller than non-OPLL group, but there is no statistical difference between the two groups (*P* > 0.05).

**Table 3:** Difference analytical results of OPLL and Non-OPLL groups

	Total	OPLL	Non-OPLL	***P*** value
The preoperative SCD, cm	1.64 ± 0.15	1.62 ± 0.13	1.65 ± 0.17	0.479
The preoperative C2–7 Cobb angle, °	12.2 ± 8.6	13.9 ± 7.5	11.7 ± 8.9	0.342
The preoperative CCI	0.09 ± 0.10	0.13 ± 0.09	0.08 ± 0.10	**0.038**
The preoperative spinal canal area, mm2	191 ± 33	186 ± 31	202 ± 38	0.135
The preoperative ROM, °	43.7 ± 11.6	43.2 ± 10.7	44.0 ± 11.8	0.275
The preoperative spinal cord area, mm2	98 ± 46	92 ± 44	97 ± 50	0.695
Door shaft position (wide: narrow)	38:45	9:10	29:35	0.876
Blood loss, ml	236 ± 177	224 ± 189	239 ± 174	0.745
Operative time, min	147 ± 38	144 ± 43	147 ± 37	0.757
Postoperative drainage volume, ml	270.0 ± 130.3	250.0 ± 123.1	273.0 ± 117.0	0.459

*OPLL* posterior longitudinal ligament, *SCD* sagittal canal diameter, *CCI* cervical curvature index, *ROM* range of motion

Besides, it is necessary for us to consider the differences in body size between men and women. To explore this factor,83 patients was divided into male group and female group to compare the anatomical parameters such as the SCD, spinal canal area and spinal cord area before surgery which can reflect the body size difference in cervical spine between the two groups. The statistical results show that the preoperative SCD, spinal canal area and spinal cord area in female group are smaller than those in male group, but there is no statistical difference between the two groups(*P* > 0.05) (Table 4).

**Table 4:** Difference analytical results of Male and Female groups

	Total	Male	Female	***P*** value
The preoperative SCD, cm	1.64 ± 0.15	1.64 ± 0.14	1.62 ± 0.13	0.510
The preoperative spinal canal area, mm^2^	191 ± 33	193 ± 35	186 ± 25	0.473
The preoperative spinal cord area, mm^2^	98 ± 46	100 ± 46	92 ± 45	0.487

*SCD* sagittal canal diameter

### Multivariate analysis

The preoperative diagnosis, door shaft position, preoperative and postoperative spinal canal area were finally included for binary Logistic regression analysis. The result showed that the preoperative diagnosis (OR = 2.076, *P* = 0.035), door shaft position (OR = 3.425, *P* = 0.020) and preoperative spinal canal area (OR = 10.217, *P* = 0.009) maybe related factors to increased spinal cord area after cervical laminoplasty with miniplate fixation (Table 5).

**Table 5:** Multivariate Analysis of Factors Associated With Increased Spinal Cord Area

	OR	95%CI	***P*** value
Diagnosis	2.076	1.054–4.090	**0.035**
Door shaft position	3.425	1.214–9.659	**0.020**
Preoperative spinal canal area	10.217	1.771–58.938	**0.009**

*CI* confidence interval, *OR* odds ratio,

*P* < 0.05 compared to the preoperative data

## Discussion

SDCL with miniplate fixation was first reported by O’Brien in 1996 and has recently become an increasingly popular method to treat multi-level cervical spondylotic myelopathy [13]. The miniplate fixation system may offer an immediate, sturdy fixation for the laminae and stabilize the cervical spinal canal expansion. At present, an increasing number of studies have focused on the dural sac. Tsuji et al. concluded that the dural sac diameter (*p* = 0.001) was significantly associated with anterior space of the spinal cord [14]. Yang et al. assessed the extent of decompression achieved by measuring the spinal canal area of the dural sac at the 3 most narrowed levels on T2-weighted magnetic resonance imaging preoperatively, 6 months postoperatively, and at final follow-up [12]. But, as we all know, the extent of increased spinal cord area is closely related to patients alongside long-term prognosis. In a recent report, the relationship between the morphological restoration of spinal cord and neurological recovery was studied, this study reveals that severe compression of the spinal cord and its greater morphological restoration during the early postoperative period affect C5 palsy development [15]. Therefore, the increased spinal cord area and the change in morphology have great clinical guiding significance. However, the current researches have not discussed or clarified the risk or protective factors that affect the spinal cord area. To solve the above problems, a multivariate analysis associated with increased spinal cord area was performed and concluded that the preoperative diagnosis, door shaft position and preoperative spinal canal area may be associated factors for increased spinal cord area following cervical laminoplasty with miniplate fixation.

Disease types affect the degree of increased spinal cord area after cervical laminoplasty. Patients diagnosed with OPLL had a greater degree of increased spinal cord area after surgery than those diagnosed with MCDH and DCSS. Aita et al. [16] observed that, although the cervical spine of patients with OPLL showed lordosis before laminoplasty, which decreased after surgery so that the cervical spine was straightened, providing physiological space for the recruitment of spinal cord area. Sugrue [17] found that laminoplasty is suitable for patients with preoperative cervical lordosis and OPLL whose spinal canal occupation rate is less than 60%. According to the above classification statistical results, the preoperative CCI was statistical difference between OPLL and other types of disease (*P* < 0.05), which proved the curvature of preoperative lordosis in OPLL group is larger than that of the non-OPLL group and provide more space for the myelocele after surgery. That would explain why patients diagnosed with OPLL had a greater degree of increased spinal cord area after surgery than those diagnosed with other diseases. However, the sample size is relatively small, and the result may be incidental due to statistical errors. Hence, a larger sample size study is needed to explore the relationship between disease types and increased in spinal cord area.

In addition, the door shaft position will also significantly affect the increased spinal cord area. In present study, patients in the wide opening group had a greater increased spinal cord area. Meanwhile, compared with the narrow opening group, the wide opening group will increase the SCD. Hatta et al [18] reported that increased the SCD were associated with an increased incidence of postoperative complications, especially C5 palsy. In patients with C5 palsy, the width of the C5 intervertebral foramen was narrower and the position of the bony gutter was wider beyond the medial part of the C5 facet joint. The distance between the lateral side of the spinal cord and bony gutter was significantly greater in patients with C5 palsy [19]. Therefore, surgeons should also consider the occurrence of postoperative complications when performing spinal cord decompression. For patients with severe spinal cord compression before surgery, door shaft position should located in the medial margin of the lateral mass to restore the spinal cord area (wide opening group); but for the patients with high-risk of postoperative C5 nerve palsy and axis pain, the door shaft position can not only ensure the decompression effect, but also restrict the spinal cord over-traction after surgery to prevent the occurrence of C5 nerve root palsy and reduce axial symptom severity effectively (narrow opening group).

In our study, the preoperative spinal canal area (*P* = 0.019) is another associated factor for increased spinal cord area. The preoperative spinal canal area essentially affects the increased the spinal cord area by affecting the anterior space of the spinal cord. The more space for the spinal cord, the better the decompression effect. In some researches, to achieve an optimal canal expansion, a canal area increase of more than 50% was necessary [20]. However, in this research, the change of the spinal canal area did not affect the results of increased spinal cord area.

The postoperative spinal canal area might be related to increased spinal cord area. Kohno et al [21] showed that widening by 95 mm^2^ in the canal area attained good outcome, suggesting that the stenotic cervical canal be enlarged to over 200 mm^2^. Hamburger et al [22] reported that patients with a spinal canal area of > 160 mm^2^ achieved a better outcome. However, some authors thought that the spinal canal area does not affect the increased the spinal cord area. In the present study, though multivariate analysis showed no significant correlation between postoperative spinal canal area and increased spinal cord area after cervical laminoplasty with miniplate fixation, univariate analysis got a significant result. This indicated us the patients whose postoperative spinal canal area might be a confounding factor to increased spinal cord area. Although gender, age, duration of symptoms, and preoperative cervical ROM were reported to be related to increased spinal cord area, our study did not confirm these relationships.

There are some limitations in our study. The present study was a retrospective study. More information may be related to the increased spinal cord area are not included, like the procedure details. The follow-up time and small sample size may lead to the loss of some potential factors related to the increased spinal cord area in the further follow-up.

## Conclusions

The clinical and radiologic data among SAI group, MAI group and LAI group after cervical laminoplasty with miniplate fixation were retrospectively analyzed, and the preoperative diagnosis, door shaft position and preoperative spinal canal area might be associated with increased spinal cord area. Therefore, the indications for cervical laminoplasty should be strictly controlled, which is suitable for patients with preoperative cervical lordosis and not suitable for OPLL whose spinal canal occupation rate is less than 60%, those patients who have kyphosis before the operation, resulting in poor decompression. During the operation, the doctor should choose the position of the door shaft according to the degree of compression of the patients’ spinal cord. It is necessary to avoid the restriction the space of the spinal cord due to narrow opening, and also to avoid a series of postoperative complications represented by C5 nerve root palsy due to wide opening.

## Data Availability

Datasets are available from the corresponding author on a reasonable request.

## Abbreviations

CCI: Cervical curvature index
CT: Computed Tomography
DCSS: Degenerative cervical spinal canal stenosis
LAI: Large spinal cord area increment
MAI: Middle spinal cord area increment
MCDH: Multiple cervical discs herniated
MRI: Magnetic resonance imaging
OPLL: Ossification of the posterior longitudinal ligament
ROM: Range of motion
SAI: Small spinal cord area increment
SCD: Sagittal canal diameter
SDCL: Single-door cervical laminoplasty
